# Development and Characterization of Cornstarch-Based Bioplastics Packaging Film Using a Combination of Different Plasticizers

**DOI:** 10.3390/polym13203487

**Published:** 2021-10-11

**Authors:** Walid Abotbina, S. M. Sapuan, M. T. H. Sultan, M. F. M. Alkbir, R. A. Ilyas

**Affiliations:** 1 Advanced Engineering Materials and Composites Research Centre, Department of Mechanical and Manufacturing Engineering, Universiti Putra Malaysia (UPM), Serdang 43400, Selangor, Malaysia; walidhmeed@gmail.com; 2 Laboratory of Biocomposite Technology, Institute of Tropical Forest and Forest Products, Universiti Putra Malaysia (UPM), Serdang 43400, Selangor, Malaysia; 3 Department of Aerospace Engineering, Universiti Putra Malaysia (UPM), Serdang 43400, Selangor, Malaysia; thariq@upm.edu.my; 4 Advanced Facilities Engineering Technology Research Cluster, Malaysian Institute of Industrial Technology (MITEC), University Kuala Lumpur, Persiaran Sinaran Ilmu, Bandar Seri Alam, Johor Bahru 81750, Johor, Malaysia; m.f.m.alkbir@gmail.com; 5 Facilities Maintenance Engineering Section, Malaysian Institute of Industrial Technology, Universiti Kuala Lumpur (UniKL), Persiaran Sinaran Ilmu, Bandar Seri Alam, Johor Bahru 81750, Johor, Malaysia; 6 School of Chemical and Energy Engineering, Faculty of Engineering, Universiti Teknologi Malaysia (UTM), Johor Bahru 81310, Johor, Malaysia; ahmadilyas@utm.my; 7 Centre for Advanced Composite Materials (CACM), Universiti Teknologi Malaysia (UTM), Johor Bahru 81310, Johor, Malaysia

**Keywords:** cornstarch, plasticizer, fructose, glycerol, film, properties

## Abstract

This work aims to develop cornstarch (CS) based films using fructose (F), glycerol (G), and their combination (FG) as plasticizers with different ratios for food packaging applications. The findings showed that F-plasticized film had the lowest moisture content, highest crystallinity among all films, and exhibited the highest tensile strength and thermostability. In contrast, G-plasticized films showed the lowest density and water absorption with less crystallinity compared to the control and the other plasticized film. In addition, SEM results indicated that FG-plasticized films had a relatively smoother and more coherent surface among the tested films. The findings have also shown that varying the concentration of the plasticizers significantly affected the different properties of the plasticized films. Therefore, the selection of a suitable plasticizer at an appropriate concentration may significantly optimize film properties to promote the utilization of CS films for food packaging applications.

## 1. Introduction

Currently, petroleum-based plastics, which are characterized by a long polymer chain, are widely used for different applications on a large scale due to their diverse mechanical properties and low cost [1,2,3,4,5]. The global production of plastics was estimated to be up to 311 million tons in 2014 [6,7], reached up to 381 million tons by 2015, and is predicted to increase four-fold by 2050 [6]. Despite the considerable contribution of petroleum-based plastics to the global economy, which exceeded billions of dollars, the non-degradability of these materials represents a huge challenge for the ecosystem, leading to many environmental crises [8,9,10,11]. It was reported that plastic waste represented approximately 79% of the waste that was discharged in landfills, recording more than 300 million tons of waste in 2015; moreover, only 12% of this waste was incinerable, and only 9% of it was recyclable [12,13].

Being recalcitrant or not biodegradable makes petroleum-based plastics one of the most critical issues to the environment currently. Thus, there is an urgent necessity to substitute other eco-friendly alternatives for these materials [14,15,16,17]. In this context, biodegradable plastics, which are naturally made from biopolymers or synthesized bio-based polymers, seem a promising alternative to replace or at least to reduce the extensive use of conventional plastics and their harmful waste [18,19,20,21,22,23]. Additionally, biopolymers derived from renewable resources such as plants and animals can play an essential role in overcoming the challenges related to the depletion of oil reserves along with the environmental issues related to the increased use of petroleum-based plastics. These biopolymers can be natural fibers, cellulose, polysaccharides, proteins, lipopolysaccharides, polyhydroxyalkanoates, or glycolipids, which are suitable in environmental applications [24,25,26,27,28,29].

Due to its ability as a linking matrix between fillers, starch is the most used biopolymer for the fabrication of biofilms with high performance and biodegradability [30,31,32,33,34,35,36]. Additionally, it is more attractive for the industrial sector because of its availability and cost-effectiveness [37]. Corn starch represents more than 85% of global starch, making the corn plant the primary source of commercial starches globally. Other plants such as wheat, rice, and potato are also important sources of native starch; however, they contribute to a minor proportion of global starch production [38]. Semi-crystalline starch represents around 70% of the mass of the corn granule, and the rest is carbohydrate, protein, oil, and ash [39,40,41]. In recent years, starch-based materials have received increased attention in different packaging applications pushed by the rising concerns about global warming [42,43,44]. Although success has been achieved in the biopolymers market, especially in the environmental aspects, this market still faces some challenges to substitute petroleum-based plastics. This is mainly due to the sensitive nature of biopolymer-based films to high moisture [45,46], and their poor mechanical properties [47,48,49]. Due to their low molecular weight and non-volatility nature, plasticizers have been widely used to improve the workability and durability of polymers since the early 1800s [50]. The primary role of these plasticizers is to weaken the attraction of hydrogen bonds in starch network amylose and amylopectin; plasticizers can also increase the mobility rate of the polymer macromolecular chain, which reduces the glass transition temperature, in turn, and enhances the flexibility and stiffness of the plasticized corn starch films [42,51,52,53,54]. Plasticizers do not only improve the physical properties of biopolymers but can also effectively enhance the processing characteristics; as a property modifier, plasticizers can decrease the second order transition temperature and the elasticity modulus, which improves the cold flexibility. Moreover, the softening effect of plasticizers helps in lowering the required processing temperature, and providing better flow properties [55]. Various types of plasticizers have been reported in the literature as adding materials for biopolymers fabrication, including fructose, glucose, and sucrose [56,57], urea [57,58], glycerol [56,59], tri-ethanolamine and glycol [57,60], as well as sorbitol and xylitol [61,62,63]. Ibrahim et al. (2019) investigated the effect of different plasticizers on corn-starch film properties, and found that film plasticized with 25% fructose produced the best set of features and achieved the highest mechanical performance [54]. In the same context, Mali et al. (2006) studied the effect of glycerol concentration (0, 20, and 40%) on corn starch and revealed that the tensile strength was reduced while the elongation at break increased with the increase in glycerol concentration [64]. In another study, Zhang et al. (2006) reported that monosaccharides (mannose, glucose, and fructose) resulted in stronger starch films (higher tensile strength). whereas polyols (glycerol and sorbitol) exhibited higher water vapor permeability [65]. Thus, the combination among different plasticizers seems attractive for optimizing the properties of CS films in different aspects. To our knowledge, no prior studies have examined the effect of combining fructose and glycerol plasticizers on the properties of CS-based film. Thus, this present study aims to investigate the potential effects of using glycerol, fructose, and a combination of glycerol and fructose at different concentrations as plasticizers on the properties of corn-starch-based bioplastics. Moreover, CS-based polymers are sensitive to humidity and have a low moisture barrier, which has limited their wider use in food applications. Because of that, special attention will be paid to optimizing the moisture barrier criteria of CS film for food packaging applications.

## 2. Materials and Methods

### 2.1. Materials

Corn starch was purchased from Thye Huat Chan Sdn. Bhd (Sungai Buloh, SGR, Malaysia), and the glycerol and fructose plasticizers were purchased from Evergreen Engineering & Resources Sdn. Bhd (Semenyih, SGR, Malaysia). The commercial starch was graded to 0.25 mm size in a sieve machine Matest A060-01 (Arcore, MB, Italy) to prepare starch powder for the characterization.

### 2.2. Preparation of Cornstarch Biopolymers

**Figure 1:**
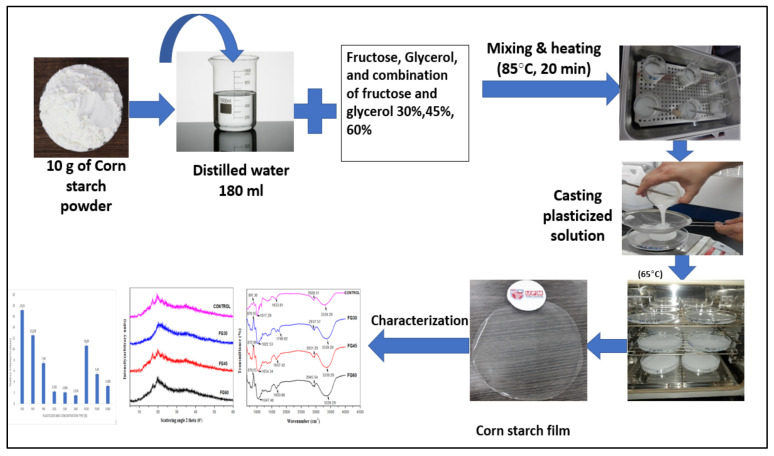
Flow chart of film preparation.

The CS-based films were prepared by the application of the solution casting technique, as depicted in Figure 1. First, both types of plasticizers were introduced into a beaker containing 180 mL of distilled water. The mixture was then heated at a temperature of 85 °C for 20 min using a water bath to prepare a homogeneous solution. After that, 10 g of corn powder was separately added into the prepared solution at different plasticizer concentrations (0, 30, 45, and 60% *w*/*w*). The solution was placed again in the water bath for 20 min at the same temperature, and the slurry was left to cool before casting on a thermal platform. The casting dishes were weighed at 45 g to ensure uniformity of film thickness. The blend was the dried for 15 h using an air circulation oven at a temperature of 65 °C. The dehydrated films were collected from the casting plates and maintained at room temperature for one week inside plastic bags before characterization. The different samples of plasticized films were coded as follows: F30%, F45%, F60% for fructose, G30%, G45%, G60% for glycerol, FG30%, FG45%, and FG60% for fructose/glycerol in a ratio of 1:1 (*v*/*v*), and CS for the control corn starch film (non-plasticized).

### 2.3. Physical Properties

#### 2.3.1. Film Density

The density of prepared films was measured using a densimeter (Mettler-Toledo (M) Sdn. Bhd., Selangor, Malaysia). Xylene was selected as a dipping solvent in this work instead of distilled water to avoid the absorption of water by the hydrophilic samples. The low density of xylene prevents the floating of prepared films. Dehydration of samples was conducted for seven days using desiccators equipped with SiO_2_ as the drying agent. The initial dry mass (m) of each sample was measured, and the biofilm was immersed in the liquid to note the volume of displaced liquid (v). The density measurement (ρ) can be calculated using Equation (1) as follows:

(1) $$\rho =m/v=g/m^{3}$$

#### 2.3.2. Film Moisture Content (MC)

To determine the content of moisture in the studied films, three samples of each film were dried for 24 h in an oven at a temperature of 105 °C. The samples were weighed before ($W_{1}$, gram) and after drying ($W_{2}$, gram) and the calculation of moisture content was performed using Equation (2) [66]:

(2) $$MC\;\left(\%\right)=\left(\frac{W_{1}-W_{2}\;}{W_{1}}\right)\times 100$$

#### 2.3.3. Film Thickness

The thickness of each film sample was measured using a digital micrometer (Mitutoyo Co., Kawasaki, Japan) with an accuracy of 0.001 mm. For more reliable results, the thickness measurement of each sample was replicated five times at different areas of the film, and the mean value of the films’ thickness was calculated. 

#### 2.3.4. Film Solubility 

To determine the film solubility, three samples (2 cm diameter) were collected from each film and dehydrated for 24 h using an oven at a temperature of 105 °C to measure the initial dry matter of each film (*W*_i_). Each sample was then immersed for 24 h in a beaker containing 30 mL of distilled water at a temperature of 23 ± 2 °C with periodic stirring. After that, the insoluble portion of the film sample was separated from the solution and dried for 24 h at 105 °C. The mass of the dried insoluble sample (*W*_0_) was used to calculate the fraction of soluble matter, which represents the solubility of the samples in water using Equation (3) as follows:

(3) $$\mathrm{Solubility}\;\left(\%\right)=\left(\frac{W_{i}-W_{0}}{W_{i}}\right)\times 100$$

#### 2.3.5. Water Absorption 

The water absorption of a material is measured by the volume of water retained by 1 g of the dehydrated material. Water absorption of the films was evaluated by a similar method reported by Ibrahim et al. [41] with little modification. One gram of the film was introduced in a pre-weighed centrifugal tube (*M*_initial_), then immersed in 25 mL of distilled water and centrifuged for 25 min at 3000 rpm. The supernatant was then removed, and the residue was dried for 30 min at 50 °C in an air circulation oven before being reweighed (*M*_final_). These steps were repeated several times until a constant mass of the sample was reached. The water absorption (WA) percentage was then calculated using Equation (4) (Ibrahim et al., 2020):

(4) $$\mathrm{WA}\;\left(\%\right)=\left(\frac{M_{\mathrm{final}}\;-{\;M}_{\mathrm{initial}}}{M_{\mathrm{initial}}}\right)\times 100$$

### 2.4. Structural Properties

#### 2.4.1. Fourier Transform Infrared Spectroscopy (FTIR)

The Fourier transform infrared (FTIR) spectroscopy analysis was conducted to test possible changes of functional groups in the films. The analysis was performed for each sample in an IR spectrometer Nicolet 6700 AEM- Thermo Nicolet Corporation (Madison, WI, USA) at 4 cm^−1^ resolution in 4000 to 500 cm^−1^ range with 42 total scans.

#### 2.4.2. X-ray Diffraction (XRD)

All of the samples were analyzed with an X-ray diffractometer 2500 (Rigaku, Tokyo, Japan) with a speed of scattering at 0.02(θ) s^−1^ over a range of angles between 5 and 60° (2θ). The operating current and voltage were fixed at 35 mA and 40 kV, respectively. The outcomes from the XRD test include relative crystallinity (Xc), crystalline area (Ic), and amorphous area (Io). Equation (5) defines relative crystallinity as a ratio between crystalline and amorphous space.

(5) $$Xc\left(\%\right)=\left(\left(Ic-Io\right)/Ic\right)\times 100$$

#### 2.4.3. Scanning Electron Microscopy (SEM)

Before conducting scanning electron microscopy (SEM), the samples were covered with a layer of gold with an argon plasma metallizer (sputter coater KK575X) (Edwards Limited, Crawley, UK) to prevent unnecessary charging. The fractured sample surface was then examined under SEM, model Hitachi S-3400N, with a 10 kV acceleration voltage.

### 2.5. Thermogravimetric Analysis (TGA)

A thermal gravimetric analysis (TGA) was performed using an analyzer Q500 V20.13 Build 39 (TA Instruments, Hüllhorst, Germany). A 10 mm sample of the film was introduced in platinum crucibles under a nitrogen atmosphere and heated starting from room temperature to a temperature of 450 °C at a rate of 10 °C/min. This test of thermal analysis assessed the thermal stability of each sample by following the mass loss over time as a function of temperature.

### 2.6. Tensile Properties

Tensile properties, including tensile strength, elongation at break, and Young’s modulus, were measured using a universal tensile machine (5 kN INSTRON, Instron, Norwood, MA, USA) to assess the mechanical behavior of the different CS film samples. The tensile machine clamps were attached to a film strip (70 to 10 mm) that was pulled at 2 mm/min crosshead speed, with an effective grip distance of 30 mm. The machine was connected to computer software (Bluehill 3), which provided the mean of each parameter using five replicates of the tested sample. 

### 2.7. Statistical Analyses

The statistical analyses of the findings were performed using Microsoft Excel 365, and the obtained data were plotted using Origin^®^ 8.5 software for the graphical presentation of the results.

## 3. Results and Discussion

### 3.1. Physical Properties

#### 3.1.1. Film Moisture Content (MC) and Density

As shown in Table 1, the results of film average moisture content using different plasticizers at different concentrations showed that the G-plasticized film had the highest moisture content followed by FG-plasticized film, whereas the lowest moisture content was observed for F-plasticized film. Moreover, a significant decrease in the moisture content was observed with the increase of F-plasticizer concentration from 30 to 60%, whereas increasing the concentration of glycerol plasticizer in the F-plasticized film from 30 to 60% significantly increased the moisture content from 11.06 to 16.97%. Similar to the G-plasticizer, the increase in the concentration of combined GF-plasticizer significantly raised the moisture content, where the moisture contents of 10.64 and 13.63% were observed for FG-plasticizer concentration of 30 and 60%, respectively. However, the observed increase in the moisture content by adding GF-plasticizer was less than the observed increase in the G-plasticized film. 

The low moisture content of F-plasticized films, when compared to glycerol-containing films, might be related to the high similarity in the molecular structure between fructose and glucose units of the polymer, which strengthened the interactions between fructose molecules and the intermolecular chains in the film [41,53]. This resulted in lower probability of the interaction between the fructose and water molecules. Meanwhile, glycerol molecules consisting of hydroxyl groups were characterized by a high water affinity, which made forming hydrogen bonds and retaining water within the matrix easier in the G-plasticized films [67,68]. Thus, fructose acted as a water-resistant agent, whereas glycerol was considered as a water-holding agent [53].

The findings presented in Table 1 show that all the plasticized biofilms had a lower density than the control biofilm: the density ranged between 1.34 and 1.49 g/cm^3^ for the plasticized films compared to a density of 1.69 g/cm^3^ observed for the control biofilm. Although the results did not show a significant difference among the selected plasticizers as the density values were too close, the lowest density was noted for G-plasticizer at a concentration of 60%. The same findings were reported by Sahari et al. (2012), who stated that density values did not demonstrate much significant difference between the various plasticizer types [69]. 

Moreover, a slight decrease in the density was observed when the concentration was increased from 20 to 60% for all plasticizers. Tarique et al. (2011) reported that this decrease in the density might be due to the association between increasing plasticizer content and the thickness of the biofilm and its volume [70]. 

#### 3.1.2. Film Thickness and Solubility

**Figure 2:**
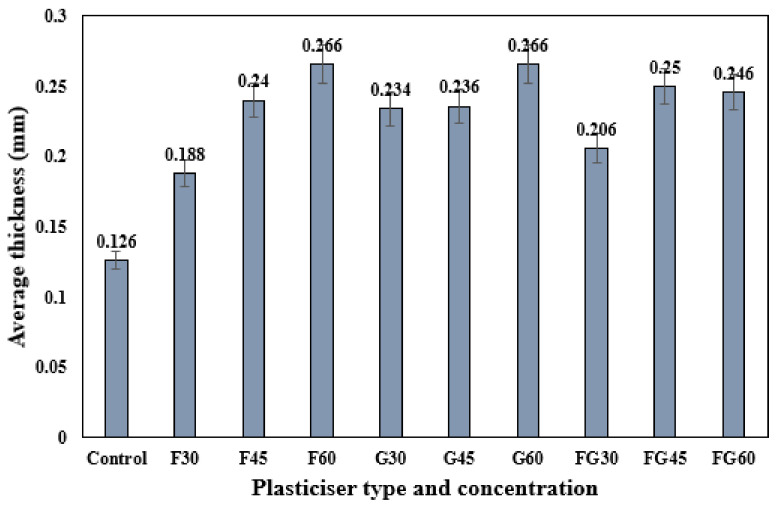
Thickness of corn starch films with various plasticizer types at different concentrations.

Figure 2 shows the thickness of different plasticized films at different plasticizer concentrations. Regardless of the type of plasticizer used, the results show that the increase of plasticizer concentration from 30 to 60% was associated with the increase in the thickness of plasticized films. Sanyang et al. (2015) reported the same findings and indicated the effect of plasticizers in the deformation of intermolecular polymer chain matrix, which provided more free volume increasing film thickness. In addition, the thickness results from varying plasticizer types showed that the thicknesses of different plasticized films were very close, although the molar mass of fructose (180 g/mole) is almost two-fold that of the molar mass of glycerol (92 g/mole). Hence, there was no significant influence of plasticizer molar mass on film thickness. This result disagreed with the findings reported in previous studies concluding that the thickness of the plasticized film was significantly related to the molar mass of the plasticizer used [42,53].

**Figure 3:**
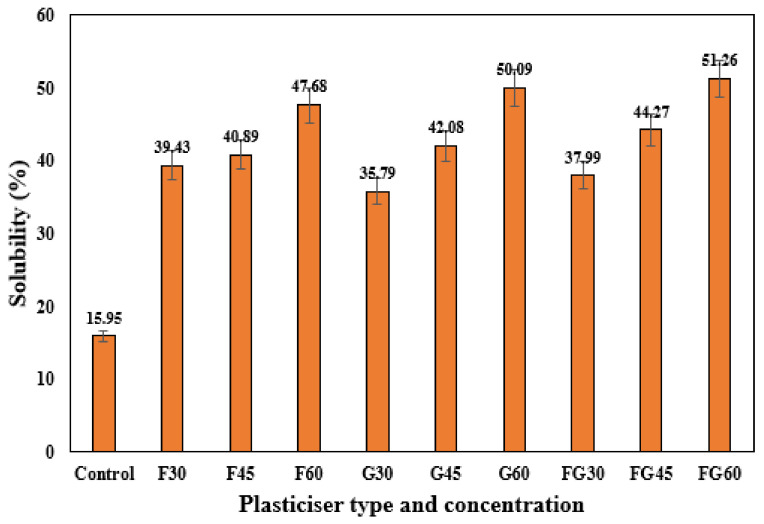
Solubility of corn starch films with various plasticizer types at different concentrations.

The water solubility of the film is also a critical property that should be considered during the characterization of biopolymers, especially for food packaging applications where water insolubility and water-resistance are sometimes required [71]. Similar to the thickness results, the same findings were observed for the solubility test of the plasticized films, where increasing the plasticizer concentration from 30 to 60% significantly increased the solubility for all types of plasticizers, as shown in Figure 3. The possible explanation of these findings is the hydrophilicity of these plasticizers as polyols contributed to weakening the bonds between polymer molecules and enlarging the free space volume in the chains. This facilitated the diffusion of water into the polymer matrix and, in turn, increased the films’ solubility [42]. The findings also indicated relatively close solubility in the films in terms of the type of plasticizer, as shown in Figure 3, where solubility ranged from 39.42 to 47.98%, 35.79 to 50.09%, and from 37.99 to 51.26% were observed for G-plasticized films, G- plasticized films, and FG-plasticized films, respectively. These similar results might be attributed to the high affinity of both glycerol and fructose to water molecules.

#### 3.1.3. Water Absorption (WA)

**Figure 4:**
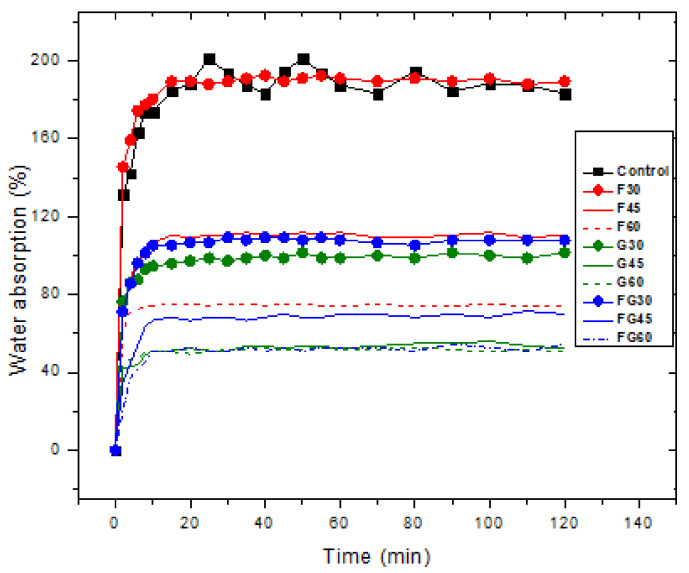
Water absorption for biofilms using different plasticizers at varying concentrations.

Water absorption ability is a critical property for starch films due to the significant role that water plays in a plasticizer. Thus, plasticized films with higher water content are characterized by higher flexibility [42,54]. In this study, the duration of the biofilms immersion in water was set at 120 min as it was reported that the plasticized samples start to dissolve in water at 140 min [72]. Figure 4 shows the results of investigating the water absorption of the plasticized films at different plasticizer concentrations. The findings show that all the films, including the control film, reached the saturation point at 40 min after the immersion, where the amount of water absorbed after this point was negligible. The highest water absorption was observed for the control sample at approximately 194.3%. Among the three studied plasticizers, F-plasticized film had the highest water absorption (187.87%), followed by FG-plasticized film, and G-plasticized film, with water absorption of 106.23 and 98.82%, respectively, at 30% of plasticizer concentration. Moreover, the findings indicated that the increase in the concentration of the plasticizers for the three types of plasticized films led to a decline in water absorption. For an increase from 30 to 60% in the plasticizer concentration, a decrease in water absorption from 187.87 to 74.10% was observed in F-plasticizer; reductions in water absorption from 98.82 to 50.58% and from 106.23 to 50.90% were recorded for FG-plasticized film and G-plasticized film, respectively. Thus, G-plasticized and FG-plasticized films possessed better water resistance than G-plasticized films. In turn, they can provide a more palatable texture and longer shelf life to high moisture products. This is understood due to the high hydrophobicity of glycerol; soluble plasticizers may block the micro-voids in the matrix of the film, causing a decrease in water absorption. At the same time, hydrophobic plasticizers can cause the formation of different phases in the produced film, which decreases the flexibility or forms discontinued areas in the matrix of the films [55].

### 3.2. Structural Properties

#### 3.2.1. Scanning Electron Microscopy (SEM)

The SEM images of the control samples exhibited a uniform and relatively smooth surface with the appearance of some non-dissolved granules related to the morphological structure of corn starch [54]. The FG- plasticized film with 30% concentration showed the smoothest surface with the absence of undissolved particles and pores (see Supplementary Materials). A relatively smooth and coherent surface without any pores was observed for the G-plasticized film at a concentration of 60%, with the presence of some impurities and agglomerated starch covering the surface. The other plasticized films, including F30, F45, F60, G30, G45, FG45, and FG60, showed less consistent surfaces with the presence of pores and microcracks in large areas of the surfaces. Although it has been reported by Edhirej et al. (2016) that F-plasticized films were evidenced to be rather smooth, coherent, and more homogeneous, the findings of the current study showed that all F-plasticized films were not smooth or homogeneous. This might be due to some differences during the preparation of the biofilm. Overall, the addition of plasticizer into CS films at appropriate concentrations led to the total dissolving of starch molecules, enhancing the coherence and integrity in the surface structure of the film [73]. The homogeneity of the matrix in the films is a good indicator of their structural integrity [74]; therefore, using a higher proportion of plasticizer produced weak and incoherent films that were hard to peel off the casting container. In contrast, preparing the film with a low concentration of plasticizer produced a film that appeared to be brittle, sticky, and difficult to remove from the casting container.

#### 3.2.2. Spectroscopy Analysis of the Film Using FTIR

**Figure 5:**
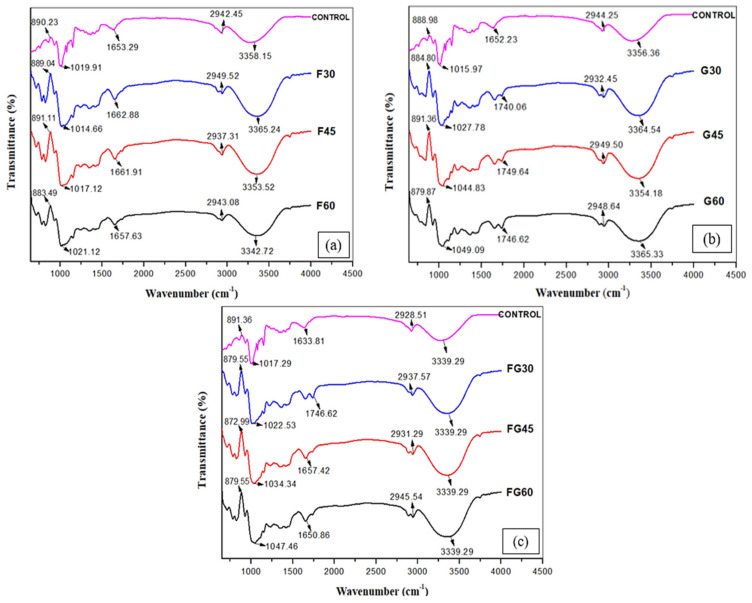
FTIR spectra of CS films with various plasticizers at different concentrations; (**a**) F-plasticized film, (**b**) G-plasticized film, (**c**) FG-plasticized film.

To identify the changes that occurred in the chemical structures of the films by adding different plasticizers at different concentrations, FTIR analysis was conducted to investigate the intermolecular rearrangement of polysaccharide chain orientation, as shown in Figure 5 [75]. The control film showed peaks at 890.23, 1019.91, 1653.21, 2942.45, and 3358.15 cm^−1^. The peak observed around 3358 cm^−1^ was associated with the stretching of the O–H groups, whereas the bands identified at 2942.45 cm^−1^ were attributed to C–H stretching. The peak at 1653.21 cm^−1^ was assigned to the bending mode of the absorbed water [76]. The characteristic peaks at 1019.91 cm^−1^ were associated with the C–O bond stretching of the C–O–C groups in the corn starch anhydroglucose ring, whereas the vibrational modes of the D-glucopyranosyl ring were around 890.23 [77,78]. Although Yin et al. [6] reported that changes in the characteristic spectral bands indicate the chemical interactions between two or more substances physically blended [79], the FTIR results in this study showed that all the plasticized films had similar spectra to the FTIR spectrum observed for the control film. These findings were in accordance with the previous study conducted by Hazrati et al. [20], who reported that the similarity observed in FTIR spectra of the control film and plasticized films was due to the fact that starch and the used plasticizers have the same functional groups, as all of them are polyols [78].

#### 3.2.3. X-ray Diffraction (XRD)

The X-ray Diffraction analysis showed that the majority of corn starch crystals were gelatinized and retrograded during the preparation of the film. The XRD analysis of the control CS-film showed four peaks at the points 15, 17.4, 19.8, and 21.8°; these findings were in agreement with the results reported by Hazrol et al. [53]. The observed peaks reflected that the control CS-film had the B-type diffraction pattern [78]. The F-plasticized film showed the same diffraction peaks observed for the control film for all the concentrations; however, a decrease in intensity of the peak at 19.8° was observed by increasing the concentration of the F-plasticizer from 30 to 60%. The addition of G-plasticizer at a concentration of 30% to the control film led to the immersion of the peak at 17.4° that appeared again when G-plasticizer was added at concentrations of 45% and 60%; meanwhile, the intensity of the peak at 19.8° increased. The FG-plasticized films at 45% and 60% of plasticizer concentrations showed a similar pattern of diffraction to the control film; however, both peaks at 17.4° and 21.8° were immersed at 25% FG-plasticized film, resulting in a large peak at 19.8° (see Supplementary Materials).

The crystallinity degree of the different samples was calculated based on the XRD pattern and are presented in Table 2. For all plasticizers, a significant increase in the crystallinity index was noticed when the plasticizer concentration was increased from 30 to 60%. Moreover, the F-plasticized film showed a higher crystallinity degree than the G- and FG-plasticized films. According to [80,81,82], there is a significant relationship between the increase in crystallinity degree of starch-based films and the decrease in the moisture content, which was compatible with the findings of this study, in which the low moisture content and the higher crystallinity degree were observed for F-plasticized films. 

### 3.3. Thermogravimetric Analysis (TGA) 

**Figure 6:**
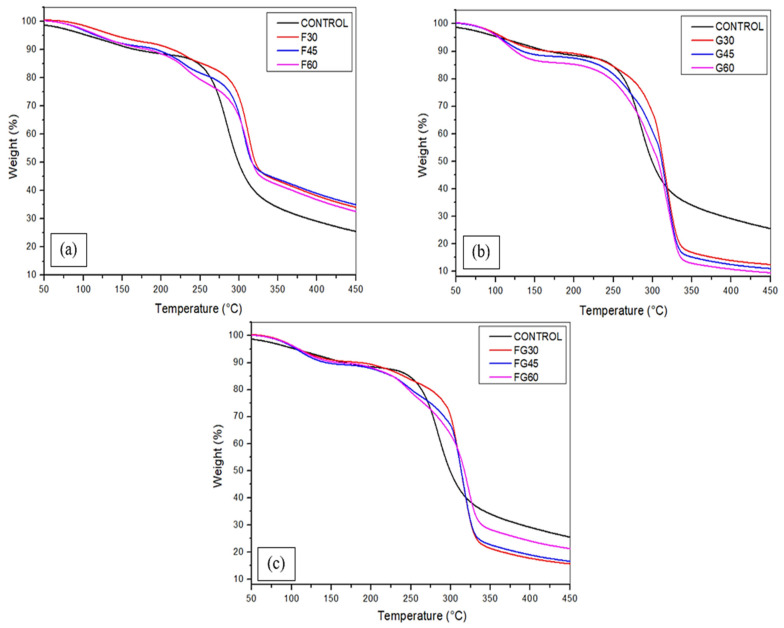
Thermogravimetric analysis of corn starch films with various plasticizers type at different concentrations: (**a**) F-plasticized film; (**b**) G-plasticized film; and (**c**) FG-plasticized film.

The thermostability of cornstarch-based biofilms was assessed using thermogravimetric analysis as represented in Figure 6 and Table 3 to determine the decomposition temperatures of each material and the fraction of material residues at the maximum degradation rate. For all the samples, three stages of degradation were recorded. The initial weight loss was observed at a temperature of below 100 °C due to the removal of a small amount of water via evaporation and dehydroxylation processes [83,84,85]. Additional heating at the range of temperature 150–200 °C led to the second loss in the film weight; the volatilization of fructose molecules and residuals was the driving process of the weight loss at this stage [86]. As illustrated in Figure 6, the weight loss of the films was limited in this stage. At the third stage of degradation, the mass loss was primarily caused by the decomposition of the water-soluble amylopectin in the films [41]. Overall, the final degradation rate of F-plasticized films ranged between 65% and 67.3%, which was very similar to the weight loss of the control sample, which recorded 65.2% as the degradation rate. The G-plasticized films showed the highest mass loss, which ranged between 87.6% and 90.5%, increasing with the concentration of plasticizers from 30 to 60%; followed by the FG-plasticized films, where the final loss of weight was estimated between 78.7% and 84.3% from the total mass. However, these results indicated that increasing the plasticizer concentration resulted in increasing the final mass loss for G-plasticized films, whereas increasing the plasticizer concentration in FG-plasticized films was associated with the decrease in the degradation rate.

### 3.4. Tensile Properties of Films

**Figure 7:**
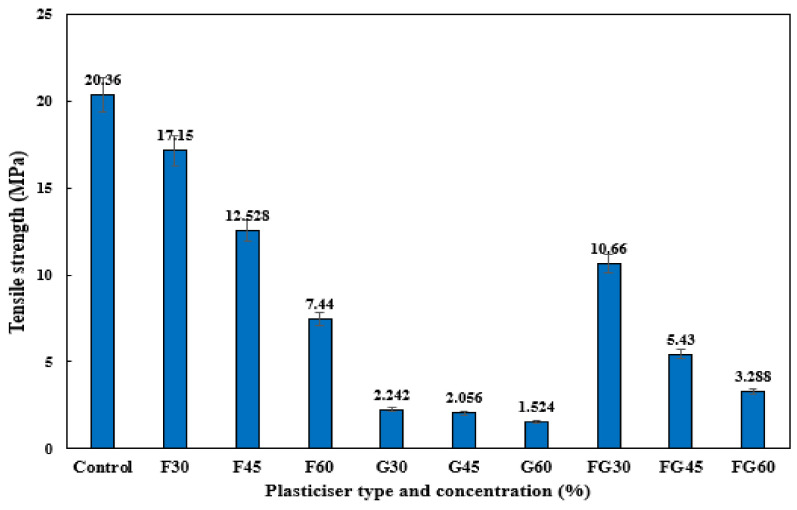
Tensile strength analysis of corn starch film with various plasticizers type at different concentrations: F30–F60: F-plasticized film; G30–G60: G-plasticized film; and FG30–FG60: FG-plasticized film.

Tensile analysis of the different plasticized films was conducted to evaluate the tensile strength, extension at the break, and Young’s modulus at different concentrations. Figure 7 demonstrates the effect of various plasticizers at different concentrations on the tensile strength of CS films. The F-plasticized film showed the highest tensile strength of 17.15 MPa for a concentration of 30%, followed by the FG plasticizer, which showed a tensile strength of 10.66 MPa at a plasticized concentration of 30%, whereas the highest tensile of G-plasticized film was 2.24 MPa. These results were consistent with the study conducted by [76], which reported the greater tensile strength of F-plasticized CS film compared to other plasticizers. For all types of plasticizers, the increase in their concentration significantly decreased the tensile strength. The increase in plasticizer concentration from 30 to 60% led to a decrease from 17.15 to 7.44 MPa in the tensile strength of F-plasticized film, a decrease from 2.24 to 1.52 MPa in the tensile strength of G-plasticized film, and a decrease from 10.66 to 3.28 MPa in the tensile strength of FG-plasticized film. Several works reported the decrease of tensile strength in the starch-based films as a response to the increase of plasticizers concentration [42,54,87]. The addition of plasticizers increased the formation of hydrogen bonds between the starch molecules and the plasticizers, causing the weakness of intramolecular interactions among the molecules of starch chains [78].

**Figure 8:**
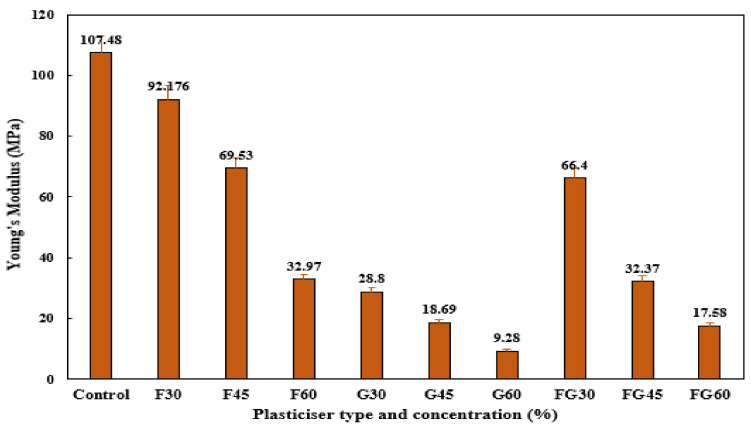
Young’s modulus of corn starch film with various plasticizer types at different concentrations: F30–F60: F-plasticized film; G30–G60: G-plasticized film; and FG30–FG60: FG-plasticized film.

Young’s modulus analysis was conducted to assess the stiffness of the films, where a high Young’s modulus reflects a high degree of stiffness of the material. As shown in Figure 8, F-plasticized films showed the highest Young’s modulus, followed by the FG-plasticized films and G-plasticized films, respectively. Among all the tested plasticizers, a decrease in tensile modulus was observed when plasticizer concentration was increased from 30 to 60% in all films, indicating that increasing plasticizer content made the films less rigid. The decrease of stiffness with increasing plasticizer content in hydrophilic films has been reported previously [88]. This behavior could be related to the structural modifications of the starch network when plasticizers were incorporated and the matrix of the films became less dense [76].

**Figure 9:**
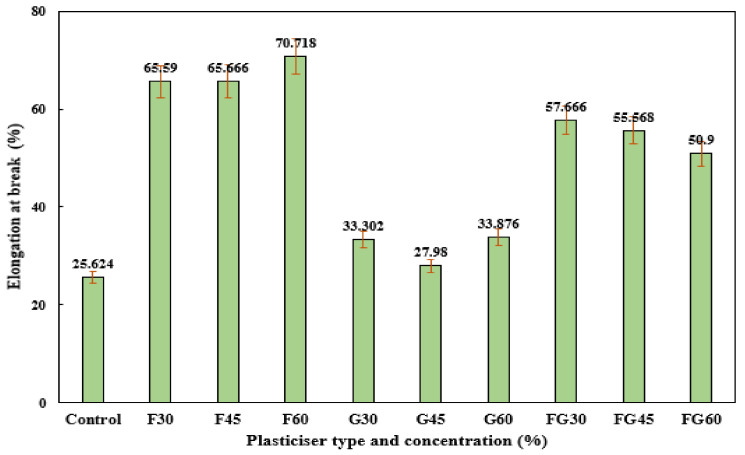
Extension at break of corn starch film with various plasticizer types at different concentrations: F30–F60: F-plasticized film; G30–G60: G-plasticized film; and FG30–FG60: FG-plasticized film.

For elongation at break, the F-plasticized films and FG-plasticized films showed higher elongations at break compared to G-plasticized films, as shown in Figure 9. However, the increase in plasticizer concentration resulted in increasing the elongation at break at F-plasticized films, whereas a decrease in elongation at break was observed in FG-plasticized films when the plasticizer concentration was increased from 30 to 60%. At the same time, the effect of plasticizer concentration on the elongation at break was not significant for G-plasticized films, where elongation of break of 33.02% and 33.87% was recorded at glycerol plasticizer concentrations of 30% and 60%, respectively. These findings were in agreement with the reported results in previous studies, which indicated the decrease of elongation break as a result of increasing plasticized concentration for F-plasticized film. Meanwhile, the opposite effect was observed when other types of plasticizers were used [76].

### 3.5. Potential of the Plasticized CS Film for Food Packaging Considering Water-Resistant Ability 

As stated earlier, the hydrophobicity of CS films resulted in high moisture content and high-water absorption as challenging factors that limit their broader use in food applications. Therefore, one of the aims of this study is to reduce the moisture content and water absorption of CS film by the incorporation of different plasticizers. Among the tested films, F-60 plasticized film exhibited the lowest moisture content, in which the incorporation of fructose plasticizer at a concentration of 60% reduced the moisture content by 47.58% compared to the control film. Additionally, G60 and FG60 biofilms showed the lowest water absorption recording 48.2% and 48.8%, respectively, compared to 74% as water absorption observed for F-60 biofilm. Moreover, the comparison of the properties of the prepared films with previous studies on CS-based films using different plastics showed the high quality of the films as water-resistant material (Table 4). 

There is no doubt that the ability of packaging materials to prevent or minimize moisture transfer between the food and the surrounding environment is a crucial property for effective food packaging [90]; however, there was a trade-off between the mechanical properties and water resistance properties of the biofilms in the present study, reflected in high water resistance biofilms with low mechanical properties and vice-versa. In this regard, FG30 biofilm, which exhibited a moderate water resistance (WC = 10.64%, WA = 103.4%), thermal resistance, as well as tensile strength (TS = 10.66 MPa) seems to be the best plasticizer as a compromise to meet the requirements of food packaging applications. 

## 4. Conclusions

In this work, the physical, structural, thermal, and mechanical properties of plasticized corn starch films were investigated to highlight the effect of plasticizer type and concentration on these properties. Incorporation of the selected plasticizers, including fructose, glycerol, and the combination of fructose/glycerol, significantly enhanced the properties of CS starch films. Moreover, the findings indicated that the addition of a specific plasticizer might enhance some properties over others. In this context, CS films containing fructose exhibited low moisture content, higher crystallinity, higher thermostability, and higher tensile strength; the G-plasticized films demonstrated the lowest density and water absorption. Thus, it seems that combining both plasticizers in FG-plasticized films effectively led to the development of corn-starch films with intermediate properties. Over the different tested plasticizers, the data indicated that the incorporation ratio of plasticizers significantly influenced the different properties of the films, which reflected the importance of determining the suitable concentration of plasticizers to optimize the quality of the film to meet the requirements of food packaging applications. In this regard, the FG30 plasticized CS film seems a promising biopolymer for food packaging applications, providing a low moisture content with a reduced water absorption capacity and good mechanical and thermal properties. Moreover, further efforts are required to investigate the chemical mechanisms of the plasticizers that affect the physical, thermal, and mechanical properties of CS starch films, which contribute to developing new techniques and methods for enhancing these properties.

## Figures and Tables

**Table 1:** Moisture content and density of biofilm at different concentrations of plasticizers.

Sample	Control	Fructose			Glycerol			Combination		
		F30	F45	F60	G30	G45	G60	FG30	FG45	FG60
MC (%)	10.15	6.11	5.40	5.32	11.06	15.19	16.97	10.64	13.31	13.63
Density (g/cm^3^)	1.69	1.49	1.46	1.4	1.40	1.36	1.34	1.45	1.41	1.39

**Table 2:** Crystallinity Index of samples using different plasticizers.

Sample	Crystallinity Index (%)
Control	21.3
F-30	33.4
F-45	36.5
F-60	37.2
G-30	20.8
G-45	19.7
G-60	19.3
FG-30	21.1
FG-45	20.6
FG-60	20.2

**Table 3:** Weight loss of all samples at different stages of degradation.

Temperature Range	Weight Loss (%)									
	C	F30	F45	F60	G30	G45	G60	FG30	FG45	FG60
20–150 °C	7.7	8.0	7.8	5.7	9.2	11.2	13.3	9.0	10.3	9.7
150–200 °C	3.8	3.5	2.7	2.9	6.3	7.4	7.9	6.3	9.1	10.8
200–500 °C	53.7	55.8	54.5	57.3	72.1	70.4	69.3	69.0	63.9	58.2
Total loss (%)	65.2	67.3	65.0	65.9	87.6	89	90.5	84.3	83.3	78.7

**Table 4:** Comparison of F-plasticized film properties with previous studies.

CS Film Type	Moisture Content (%)	Water Absorption (%)	Mechanical Properties			
			Tensile Strength (MPa)	Tensile Modulus (MPa)	Elongation at Break (%)	Ref.
Control	10.15	194.3	20.36	107.48	25.62	This study
Fructose	5.32 (F60)	74.1(F60)	7.44 (F60)	32.97 (F60)	70.718 (F60)	This study
Glycerol	11.06 (G30)	48.2 (G60)	2.24 (G30), 1.52 (G60)	28.8 (G30), 9.28 (G60)	33.30 (G30), 33.88 (G60)	This study
Fructose/Glycerol	10.64 (FG30)	48.8(FG60)	10.66 (FG30), 3.29 (FG60)	66.4 (FG30), 17.58 (FG60)	57.67 (FG30), 50.90 (FG60)	This study
Sorbitol	9.25–10.04	147	13.62 (S30)	495.97 (S30)	NA	[53]
Glycerol	14.7–16.55	112	2.53 (G30)	19.43 (G30)	NA	[53]
Sorbitol/glycerol	9.11–14.99	135	5.74 (SG30)	47.17 (SG30)	NA	[53]
Urea	21.05–27.86	NA	0.62 (U25)	1.67 (U25)	NA	[54]
Formamide	NA	96.09	3.42	NA	105.21	[89]
Ethylene Glycol	NA	92.24	3.8	NA	98.22	[89]

## Data Availability

Not applicable.

## Supplementary Materials

The following are available online at https://www.mdpi.com/article/10.3390/polym13203487/s1, Figure S1: Scanning electron micrograph of CS films with various plasticizers and concentrations, Figure S2: XRD analysis of corn starch film with various plasticizers type at different concentrations; (a) F-plasticized film, (b) G-plasticized film, (c) FG-plasticized film.
