# Supplementary material for: Development and Characterization of Cornstarch-Based Bioplastics Packaging Film Using a Combination of Different Plasticizers
Source: Polymers (Basel). 2021 Oct 11;13(20):3487. doi: 10.3390/polym13203487 (PMC8539400; doi:10.3390/polym13203487)
Supplement: Supplementary file 1 [file polymers-13-03487-s001.zip › polymers-1366716-supplementary.pdf]

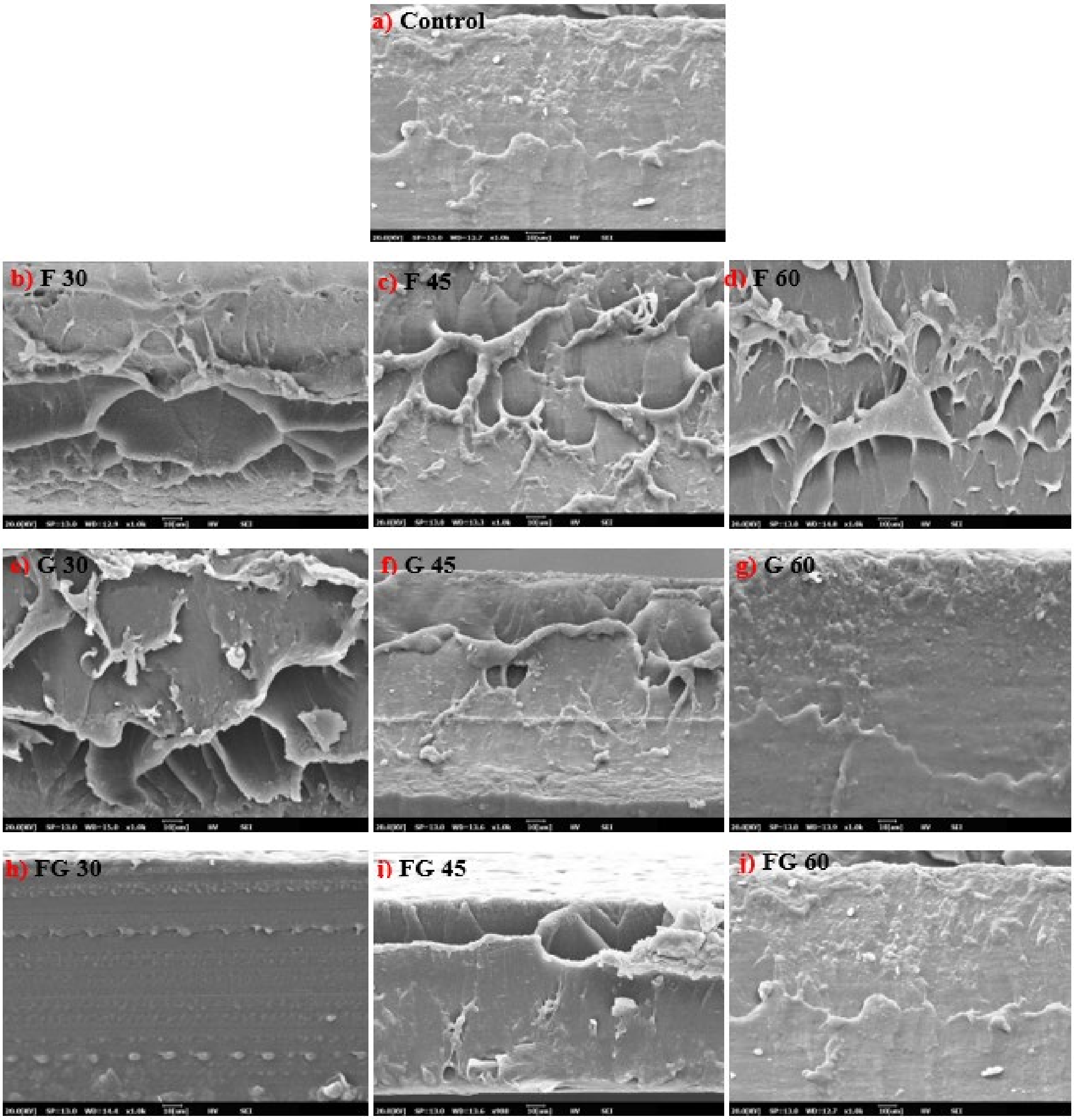

Figure S1. Scanning electron micrograph of CS films with various plasticizers and concentrations.

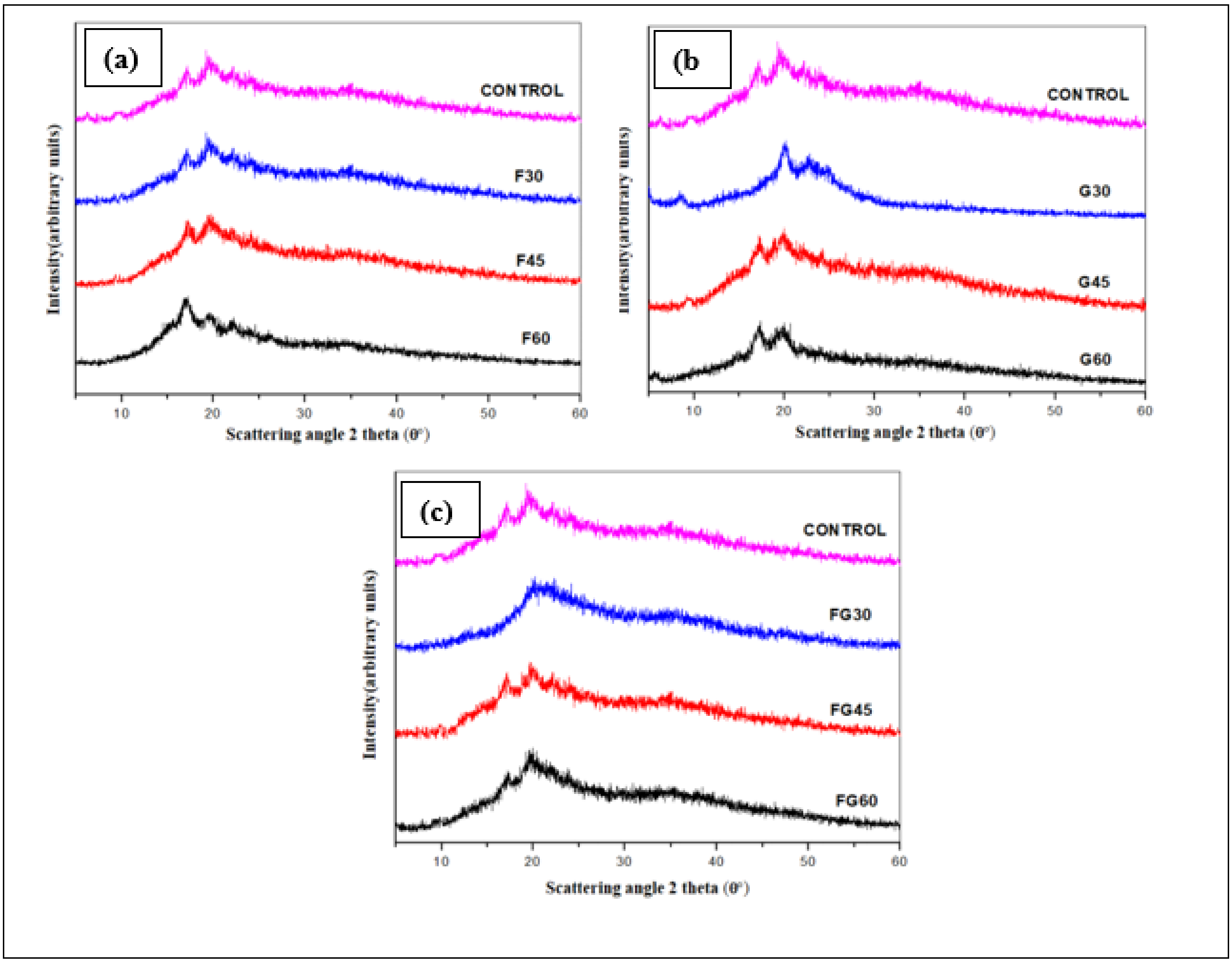

Figure S2. XRD analysis of corn starch film with various plasticizers type at different concentrations; (a) F-plasticized film, (b) G-plasticized film, (c) FG-plasticized film
